# Supplementary material for: Factors related to willingness to participate in biomedical research on neglected tropical diseases: A systematic review
Source: PLoS Negl Trop Dis. 2024 Mar 12;18(3):e0011996. doi: 10.1371/journal.pntd.0011996 (PMC10978008; doi:10.1371/journal.pntd.0011996)
Supplement: S1 Appendix — (PDF) [file pntd.0011996.s001.pdf]

To enable PROSPERO to focus on COVID-19 submissions, this registration record has undergone basic automated checks for eligibility and is published exactly as submitted. PROSPERO has never provided peer review, and usual checking by the PROSPERO team does not endorse content. Therefore, automatically published records should be treated as any other PROSPERO registration. Further detail is provided here.

Citation

Vinicius Silva, João Rezende, Caio Marques, Martha Silveira, Mitermayer Reis. Factors related to willingness to participate in biomedical research on neglected tropical diseases: Systematic Review. PROSPERO 2020 CRD42020212536 Available from:
https://www.crd.york.ac.uk/prospero/display_record.php?ID=CRD42020212536

Review question

The aim of the review is to increase the understanding of: a) What are the reasons for and against participating in biomedical research on neglected disease? b) What are the enablers and barriers for participating in biomedical research on neglected disease? c) Do recruitment strategies influence in willingness to participate in biomedical research on neglected disease?

Searches

Studies for review were identified from searches of the following academic databases:

MEDLINE/PubMed (07/01/2020), Embase (08/03/2020), Web of Science (09/08/2020) and Index Medicus Global (08/27/2020). Supplementary searches will be done in Google Scholar, snowballing of included studies and hand searching references of included studies. No restriction.

Types of study to be included

Review will include clinical trials, longitudinal cohorts, cross-sectional study, qualitative study as focus group discussions (FGDs), semistructured interviews and in-depth interviews.We will include only English, Spanish and Portuguese papers and articles that have an available full-text. Moreover, we will exclude the following types of manuscripts: conference papers, reviews, notes, case reports, letters to the editor, editor's notes, extended abstracts, proceedings, patents, editorials, and other editorial materials.

Condition or domain being studied

Recruitment is an essential step of research with human subjects. Recuse to participate in research is an unpleasant experience for researchers that difficult conducting trials.

Primary and secondary researches have investigated barriers and enablers to participate in biomedical research, especially in low-and middle-income countries. However, there are no reviews that focus specifically on reasons why people refuse to participate in biomedical research on neglected disease. Taking in account that tropical neglected diseases are more prevalent in socioeconomically vulnerable communities, it may be numerous and different factors that may limit the efficacy of recruitment.

Participants/population

Inclusion: People who are potential candidates to take part in biomedical research on neglected tropical disease.

People who were invited to take part in biomedical research on neglected tropical disease

People who were asked if they would take part in biomedical research on neglected tropical disease.

Exclusion: Not applicated

Intervention(s), exposure(s)

Not applicable

Comparator(s)/control

Not applicable

Context

The World Health Organization (WHO) defined Neglected Tropical Diseases (NTD) as a varied group of communicable diseases that prevail in tropical and subtropical, affect approximately one billion people and cost developing economies billions of dollars every year.

WHO list in NTD portfolio 25 diseases: Buruli ulcer, Chagas disease, Chikungunya, Chromoblastomycosis, Cysticercosis, Dengue fever, Dracunculiasis, Echinococcosis, Foodborne trematodiases, Human African trypanosomiasis, Leishmaniasis, Leprosy, Lymphatic filariasis, Mycetoma, Onchocerciasis, Rabies, Scabies and other ectoparasites, Schistosomiasis, Soil-transmitted helminthiases, Snakebite envenoming, Trachoma, Taeniasis, Yaws.

Most NTDs occur in remote areas of low- and middle-income countries and conducting research in those settings signifie fight specific challenges that are linked to the social-ecological systems, infrastructure (or lack thereof), culture, ethics requirements, and conflicting health policies, among others. In this context marked for poverty and social exclusion, numerous factors may limit the efficacy of the informed consent process for biomedical research on NTD and, unfortunately, lead to exclusion from medical research, which is most often crucially needed to address their health problems.

Biomedical research on NTD has been underfunded compared with other diseases such as malaria, human immunodeficiency virus and tuberculosis. This situation increases the need to use better financial and human resources and a possible solution to this is to understand barriers and enablers for participating in biomedical research on neglected disease.

Main outcome(s)

Primary outcomes are reasons for and against participating in biomedical research on neglected diseases research. We will classify these reasons according to frequencies viewed in primary researches and we will analyze the relative importance of reasons compared to others. Measures of effect

Not applicable

Additional outcome(s)

Secondary outcomes are: a) the influence of recruitment strategies in willingness to participate in biomedical research on neglected tropical disease; b) the influence of urban or rural location in willingness to participate in biomedical research on neglected tropical disease; c) the difference in reason for and against participating in biomedical research among neglected tropical diseases

Measures of effect

Not applicated

Data extraction (selection and coding)

Selection of studies:

First, the selection of articles was carried out by reading titles and abstracts by two independent reviewers, who were not be blinded to the titles of the journals or to the authors or institutions of the study. Then, a complete reading of the eligible items was carried out in the first stage. Disagreements will be resolved by consensus between the reviewers. Eligibility for review and the reason for exclusion will be indicated.

Data extraction and management:

Data extraction will be done independently by three independent reviewers, using a form adapted for this systematic review. Items for data extraction will include “authors, year of publication, country, aims, neglected disease, research question, urban/rural location, recruitment context, data analysis, sample, barriers for participating, enablers for participating, author’s conclusion and discussion”. We will use Excel to organize items from data extraction. A pilot test of the data abstraction form and the review process will be conducted on 3 articles before starting the review.

Risk of bias (quality) assessment

Because of the nature of the research question for this review, we have decided to include studies regardless of the study design. Therefore, we will not pretend to perform the risk of bias assessment.

Strategy for data synthesis

Two reviewers will systematically read the selected studies and analyze the results of each of them. We pretend to create an overall ranking of the frequency of reasons for participation and non-participation and the relative importance of reasons compared to others.

Ranking of reasons will be stratified for the following categories, if available in more than one article: urban vs. rural, Neglected Tropical diseases, World regions and hypothetical (i.e. empirical studies that ask participants about potential participation in studies that do not (yet) exist or enrol participants) vs. ‘real’ studies.

We also describe results in a narrative way separating the results into two topics: reasons for and against participating.

Analysis of subgroups or subsets

There will be a subgroup analysis of each neglected disease. The factor associated with the refusal to participate in research for one disease may be different from another.

Contact details for further information

Vinicius Raimundo Santos da Silva
viniciusraimundosilva@gmail.com

Organisational affiliation of the review

Federal University of Bahia
http://www.fameb.ufba.br/

Review team members and their organisational affiliations

Mr Vinicius Silva. Faculty of Medicine of Bahia, Federal University of Bahia-Salvador, Bahia, Brazil
Mr João Rezende. Faculty of Medicine of Bahia, Federal University of Bahia-Salvador, Bahia, Brazil
Mr Caio Marques. Faculty of Medicine of Bahia, Federal University of Bahia-Salvador, Bahia, Brazil
Dr Martha Silveira. Gonçalo Moniz Institute, Oswald Cruz Foundation, Ministry of Health-Salvador, Bahia, Brazil
Dr Mitermayer Reis. Faculty of Medicine of Bahia, Federal University of Bahia-Salvador, Bahia, Brazil; Gonçalo Moniz Institute, Oswald Cruz Foundation, Ministry of Health-Salvador, Bahia, Brazil; Department of Epidemiology of Microbial Diseases, School of Public Health, Yale University - New Haven, Connecticut, United States of America Department of Epidemiology of Microbial Diseases, School of Public Health, Yale University - New Haven, Connecticut, United States of America

Type and method of review

Epidemiologic, Systematic review

Anticipated or actual start date

26 April 2020

Anticipated completion date
10 September 2021

Funding sources/sponsors
The review has been funded by a Federal University of Bahia (UFBA),

Gonçalo Moniz Institute (Oswald Cruz Foundation) and The Brazilian National Council for Scientific and Technological Development (CNPq). The funders played no active role in the data collection, extraction and analysis.

Conflicts of interest

Language
English

Country
Brazil

Stage of review
Review Ongoing

Subject index terms status
Subject indexing assigned by CRD

Subject index terms
MeSH headings have not been applied to this record

Date of registration in PROSPERO
13 November 2020

Date of first submission
13 October 2020

Stage of review at time of this submission

Stage	Started	Completed
Preliminary searches	Yes	Yes
Piloting of the study selection process	Yes	Yes
Formal screening of search results against eligibility criteria	Yes	Yes
Data extraction	No	No
Risk of bias (quality) assessment	No	No
Data analysis	No	No

The record owner confirms that the information they have supplied for this submission is accurate and complete and they understand that deliberate provision of inaccurate information or omission of data may be construed as scientific misconduct.

The record owner confirms that they will update the status of the review when it is completed and will add publication details in due course.

Versions

13 November 2020

Powered by TCPDF (www.tcpdf.org)
