# Supplementary material for: Factors related to willingness to participate in biomedical research on neglected tropical diseases: A systematic review
Source: PLoS Negl Trop Dis. 2024 Mar 12;18(3):e0011996. doi: 10.1371/journal.pntd.0011996 (PMC10978008; doi:10.1371/journal.pntd.0011996)
Supplement: S3 Appendix — (DOCX) [file pntd.0011996.s003.docx]

**S3 Appendix**: Databases Search strategies

| **DATABASE** | **SEARCH STRATEGY** |
| --- | --- |
| **MEDLINE/PUBMED**  **Date run**:  June 7th, 2020 | 1. **TERMS RELATED TO TYPES OF CLINICAL RESEARCH:**   "Biomedical Research" [MESH] OR "research subjects"[MeSH] OR "Research Design"[MeSH] OR "Community-Based Participatory Research"[Mesh] OR "Patient Selection"[MESH] OR "clinical trial"[Publication Type] OR "clinical trials as topic"[MeSH Terms] OR “clinical trial”[TIAB] OR trial*[TIAB] O=R “Biomedical Research”[TIAB] OR “health research studies” OR “research design” OR “Medical research” OR “stored human biological materials”[TIAB] OR “stored human samples”[TIAB] OR Survey[TIAB] OR “Community-based participatory research”[TIAB] OR "observational study"[Publication Type] OR "observational studies as topic"[MeSH Terms] OR Observational stud*[TIAB] OR Population Bank*[TIAB] OR "population groups"[MeSH Terms] OR “cohort studies"[MeSH Terms] OR Cohort[TIAB] OR “longitudinal studies"[MeSH Terms] OR Longitudinal stud*[TIAB] OR clinical stud*[TIAB] OR intervention stud*[TIAB] OR descriptive stud*[TIAB] OR "focus groups"[MeSH Terms] OR “Focus Group” OR "feasibility studies"[MeSH Terms] OR “Feasibility Studies” OR "surveys and questionnaires"[MeSH Terms] OR “Questionnaires”[TW]   1. **TERMS RELATED TO COMMUNITY PARTICIPATION IN RESEARCH**   "Cooperative Behavior"[Mesh] OR "Community Participation"[MESH] OR "Informed Consent"[MESH] OR “Community-Institutional Relations”[MeSH] OR “Patient Participation”[MESH] OR "Researcher-Subject Relations"[MeSH] OR "Vulnerable Populations"[MESH] OR “Human Subject Research”[TIAB] OR “Human Subjects  Research”[TIAB] OR “Community Consultation”[TIAB]  OR recruit*[TIAB]  OR Enroll*[TIAB]  OR “Public Involvement” OR “public and patient involvement” OR PPI OR “patient selection” OR “Community Participation” OR “participation in biomedical research”[TIAB] OR “participation in Clinical Trials” OR “Health Research Participation” OR “community engagement”[TIAB] OR “trial participation”[TIAB] OR “research participation”[TIAB] OR “patient participation”[TIAB] OR “patient recruitment”[TIAB] OR “patient involvement” OR “patient engagement”[TIAB] OR "participatory research" OR "participatory action" OR "Health Education"[Mesh] OR "Attitude to Health"[Mesh]   1. **TERMS RELATED TO WILLINGNESS OR UNWILLINGNESS TO PARTICIPATE IN RESEARCH**   "Refusal to Participate"[MESH] OR "Motivation"[MeSH] OR “Willingness to participate”[TIAB] OR “Motivation to participate”[TIAB] OR “attitudes towards research”[TIAB] OR “motivate to consent”[TIAB] OR “motivating factor to participate”[TIAB] OR “willing to consent”[TIAB] OR “participation and retention”[TIAB] OR “Reasons to participate”[TIAB] OR “Facilitators to participate”[TIAB] OR “Trial preparedness”[TIAB] OR “Interest in participation”[TIAB] OR “Agree to participate”[TIAB] OR “Refusal to participate”[TIAB]  OR “Refusing Participation” [TIAB] OR “Barriers to participation”[TIAB] OR “barriers to participate”[TIAB] OR “unwilling to participate”[TIAB] OR “challenges to participation”[TIAB] OR “concerns regarding participation”[TIAB] OR “Participants refusal” [TIAB]  OR “refuse to join” [TIAB]  OR “refuse to participate” [TIAB] OR “Motivational Factors” [TIAB] OR “knowledge and perceptions of clinical trials”[TIAB] OR preparedness[TIAB] OR “recruitment and retention”[TIAB] OR “difficult recruitment” [TIAB] OR “difficult to reach” [TIAB] OR “hard to reach” [TIAB] OR Stakeholder*[TW] OR "Stakeholder Participation"[Mesh]   1. **TERMS RELATED TO RESEARCH ON NEGLECTED TROPICAL DISEASES**   “Buruli ulcer”[Mesh] OR “Buruli ulcer” OR "Chagas Disease"[Mesh] OR “Chagas disease” OR "dengue"[Mesh] OR “Dengue”[TIAB] OR "dracunculiasis"[Mesh] OR "dracunculiasis” OR “Guinea-worm disease” OR “GWD”[TIAB] OR "echinococcosis"[MeSH] OR "echinococcosis” OR "Trypanosomiasis, African"[Mesh] OR “trypanosomiasis” OR “Trypanosomiases” OR "Leishmaniasis"[MeSH] OR “Leishmaniasis” OR “Leishmaniases” OR “Leishmania Infections” OR "leprosies" OR "leprosy"[MeSH] OR "leprosy" OR "mycetoma"[MeSH] OR "mycetoma" OR "Elephantiasis, Filarial"[Mesh] OR “Lymphatic filariasis” OR “Elephantiasis” OR “Elephantiasis” OR “filariasis” OR “Filariasis” OR "Onchocerciasis"[Mesh] OR “Onchocerciasis” OR "Rabies"[Mesh] OR “Rabies” OR Lyssa OR “Hydrophobia” OR "Schistosomiasis"[MeSH] OR “Schistosomiasis” OR Bilharziasis OR “Katayama Fever” OR “Schistosoma Infection” OR “Soil-transmitted helminthiases” OR (“Soil-transmitted” AND “helminthiasis”) OR “helminthiasis"[MeSH] OR “helminthiases” OR “helminthiasis” OR “Trachoma”[Mesh] OR “Trachoma” OR “trachomas” OR “Egyptian Ophthalmia” OR “Teniasis” OR “Taeniasis”[MeSH] OR “taeniasis” OR “Taenia Infection” OR “Yaws”[MeSH] OR “yaws” OR Frambesia OR “Tropical Medicine” OR “Tropical Medicine”[MeSH] OR "Neglected Diseases"[MeSH] OR “Neglected Diseases” OR “parasitic infections”[TIAB] OR “Trematode infections”[Mesh] OR “trematode infections”[TIAB] OR “Fascioliasis”[Mesh] OR “Fascioliasis” OR “Clonorchiasis”[MeSH] OR “Clonorchiasis” OR “Opisthorchiasis”[MeSH] OR “Opisthorchiasis” OR "Neurocysticercosis"[Mesh] OR “Neurocysticercosis” OR “Chikungunya Fever”[MeSH] OR “Chikungunya”[TIAB] OR “Snakebite envenoming” OR "Scabies"[MeSH] OR "scabies" OR “Ectoparasitic Infestations”[MeSH] OR “Ectoparasitic Infestations” OR “Chromoblastomycosis”[MeSH] OR “chromoblastomycosis” OR “Cysticercosis”[MeSH] OR “Cysticercosis"  **1 AND 2 AND 3 AND 4**  453 results (2020) 582 (27/04/2023) |
| **EMBASE**  Embase.com by Elsevier  **Date run**:  Aug 3th, 2020 | 1. **TERMS RELATED TO TYPES OF CLINICAL RESEARCH:**   'medical research'/exp OR 'research subject'/exp OR 'participatory research'/exp OR 'patient selection'/exp OR 'clinical trial (topic)'/exp OR 'controlled clinical trial (topic)'/exp OR 'clinical development plan'/exp OR 'research'/exp OR 'clinical trial':ti,ab OR trial*:ti,ab OR 'biomedical research':ti,ab OR 'medical research':ti,ab OR 'health research studies' OR 'research design' OR 'stored human biological materials':ti,ab OR 'stored human samples':ti,ab OR survey:ti,ab OR 'community-based participatory research':ti,ab OR 'observational stud*':ti,ab OR 'population bank*':ti,ab OR 'population group'/exp OR 'cohort analysis'/exp OR cohort:ti,ab OR 'longitudinal study'/exp OR 'longitudinal stud*':ti,ab OR 'clinical stud*':ti,ab OR 'intervention stud*':ti,ab OR 'descriptive stud*':ti,ab OR 'focus group' OR 'feasibility study'/exp OR 'feasibility studies' OR 'questionnaire'/exp OR questionnaire*:ti,ab,de,tn   1. **TERMS RELATED TO COMMUNITY PARTICIPATION IN RESEARCH**   'cooperation'/exp OR 'community participation'/exp OR 'informed consent'/exp OR 'patient participation'/exp OR 'vulnerable population'/exp OR 'human subject research':ti,ab OR 'human subjects research':ti,ab OR 'community consultation':ti,ab OR recruit*:ti,ab OR enrol*:ti,ab OR 'public involvement' OR 'public and patient involvement' OR ppi OR 'patient selection' OR 'community participation' OR 'participation in biomedical research':ti,ab OR 'participation in clinical trials' OR 'health research participation' OR 'community engagement':ti,ab OR 'trial participation':ti,ab OR 'research participation':ti,ab OR 'patient participation':ti,ab OR 'patient recruitment':ti,ab OR 'patient involvement' OR 'patient engagement':ti,ab OR 'participatory research' OR 'participatory action' OR stakeholder*:ti,ab,de,tn OR 'stakeholder engagement'/exp OR 'health education'/exp OR 'attitude to health'/exp   1. **TERMS RELATED TO WILLINGNESS OR UNWILLINGNESS TO PARTICIPATE IN RESEARCH**   'refusal to participate'/exp OR 'motivation'/exp OR 'willingness to participate':ti,ab OR 'motivation to participate':ti,ab OR 'attitudes towards research':ti,ab OR 'motivate to consent':ti,ab OR 'motivating factor to participate':ti,ab OR 'willing to consent':ti,ab OR 'participation and retention':ti,ab OR 'reasons to participate':ti,ab OR 'facilitators to participate':ti,ab OR 'trial preparedness':ti,ab OR 'interest in participation':ti,ab OR 'agree to participate':ti,ab OR 'refusal to participate':ti,ab OR 'refusing participation':ti,ab OR 'barriers to participation':ti,ab OR 'barriers to participate':ti,ab OR 'unwilling to participate':ti,ab OR 'challenges to participation':ti,ab OR 'concerns regarding participation':ti,ab OR 'participants refusal':ti,ab OR 'refuse to join':ti,ab OR 'refuse to participate':ti,ab OR 'motivational factors':ti,ab OR 'knowledge and perceptions of clinical trials':ti,ab OR preparedness:ti,ab OR 'recruitment and retention':ti,ab OR 'difficult recruitment':ti,ab OR 'difficult to reach':ti,ab OR 'hard to reach':ti,ab   1. **TERMS RELATED TO RESEARCH ON NEGLECTED TROPICAL DISEASES**   'buruli ulcer'/exp OR 'buruli ulcer' OR 'chagas disease'/exp OR 'chagas disease' OR 'dengue'/exp OR dengue:ti,ab OR 'dracunculiasis'/exp OR dracunculiasis OR 'guinea-worm disease' OR gwd:ti,ab OR 'echinococcosis'/exp OR echinococcosis OR 'african trypanosomiasis'/exp OR trypanosomiasis OR trypanosomiases OR 'leishmaniasis'/exp OR leishmaniasis OR leishmaniases OR 'leishmania infections' OR leprosies OR 'leprosy'/exp OR leprosy OR 'mycetoma'/exp OR mycetoma OR 'lymphatic filariasis'/exp OR 'lymphatic filariasis' OR elephantiasis OR filariasis OR 'onchocerciasis'/exp OR onchocerciasis OR 'rabies'/exp OR rabies OR lyssa OR hydrophobia OR 'schistosomiasis'/exp OR schistosomiasis OR bilharziasis OR 'katayama fever' OR 'schistosoma infection' OR 'soil-transmitted helminthiases' OR ('soil transmitted' AND helminthiasis) OR 'helminthiasis'/exp OR helminthiases OR helminthiasis OR 'trachoma'/exp OR trachoma* OR 'egyptian ophthalmia' OR teniasis OR taeniasis OR 'taenia infection' OR 'yaws'/exp OR yaws OR frambesia OR 'tropical medicine' OR 'tropical medicine'/exp OR 'neglected disease'/exp OR 'neglected diseases' OR 'parasitic infections':ti,ab OR trematodiasis OR 'trematode infections':ti,ab OR 'fascioliasis'/exp OR fascioliasis OR 'clonorchiasis'/exp OR clonorchiasis OR 'opisthorchiasis'/exp OR opisthorchiasis OR 'neurocysticercosis'/exp OR neurocysticercosis OR 'chikungunya'/exp OR chikungunya:ti,ab OR 'snakebite envenoming' OR 'snakebite'/exp OR 'snake venom'/exp OR 'scabies'/exp OR scabies OR 'ectoparasitosis'/exp OR 'ectoparasitic infestations' OR 'chromomycosis'/exp OR chromoblastomycosis OR 'cysticercosis'/exp OR cysticercosis  **1 AND 2 AND 3 AND 4**  355 results (2020) 806 (27 de abril 2023) |
| **WEB OF SCIENCE**  (Core Collection by Clarivate Analytics)  **Date run**:  Aug 9th, 2020 | 1. **TERMS RELATED TO TYPES OF CLINICAL RESEARCH:**   ("medical research"OR "Biomedical Research" OR "research subjects" OR "Research Design" OR "Community-Based Participatory Research" OR "Patient Selection" OR "clinical trial" OR trial* OR "Biomedical Research" OR "health research studies" OR "research design" OR "Medical research" OR "stored human biological materials" OR "stored human samples" OR Survey OR "Community-based participatory research" OR "observational study" OR "observational studies as topic" OR "Observational stud*" OR "Population Bank*" OR "population groups" OR "cohort studies" OR Cohort OR "longitudinal studies" OR "Longitudinal stud*" OR "clinical stud*" OR "intervention stud*" OR "descriptive stud*" OR "focus groups" OR "Focus Group" OR "feasibility studies" OR "Feasibility Studies" OR "surveys and questionnaires" OR Questionnaires)   1. **TERMS RELATED TO COMMUNITY PARTICIPATION IN RESEARCH**   "Buruli ulcer" OR "Chagas disease" OR dengue OR dracunculiasis OR "Guinea-worm disease" OR GWD OR echinococcosis OR "African trypanosomiasis" OR trypanosomiasis OR Trypanosomiases OR Leishmaniasis OR Leishmaniases OR "Leishmania Infections" OR leprosies OR leprosy OR mycetoma OR "Lymphatic filariasis" OR Elephantiasis OR filariasis OR Onchocerciasis OR Rabies OR Lyssa OR Hydrophobia OR Schistosomiasis OR Bilharziasis OR "Katayama Fever" OR "Schistosoma Infection" OR "Soil-transmitted helminthiases" OR (Soil-transmitted AND helminthiasis) OR helminthiasis OR helminthiases OR Trachoma* OR "Egyptian Ophthalmia" OR Teniasis OR taeniasis OR "Taenia Infection" OR yaws OR Frambesia OR "Tropical Medicine" OR "Neglected Diseases" OR "parasitic infections" OR trematodiasis OR "trematode infections" OR Fascioliasis OR Clonorchiasis OR Opisthorchiasis OR Neurocysticercosis OR Chikungunya OR "Snakebite envenoming" OR snakebite OR “snake venom” OR scabies OR ectoparasitosis OR "Ectoparasitic Infestations" OR chromomycosis OR chromoblastomycosis OR Cysticercosis   1. **TERMS RELATED TO WILLINGNESS OR UNWILLINGNESS TO PARTICIPATE IN RESEARCH**   "Refusal to Participate" OR Motivation OR "Willingness to participate" OR "Motivation to participate" OR "attitudes towards research" OR "motivate to consent" OR "motivating factor to participate" OR "willing to consent" OR "participation and retention" OR "Reasons to participate" OR "Facilitators to participate" OR "Trial preparedness" OR "Interest in participation" OR "Agree to participate" OR "Refusing Participation" OR "Barriers to participation" OR "barriers to participate" OR "unwilling to participate" OR "challenges to participation" OR "concerns regarding participation" OR "Participants refusal" OR "refuse to join" OR "refuse to participate" OR "Motivational Factors" OR "knowledge and perceptions of clinical trials" OR preparedness OR "recruitment and retention" OR "difficult recruitment" OR "difficult to reach" OR "hard to reach"   1. **TERMS RELATED TO RESEARCH ON NEGLECTED TROPICAL DISEASES**   Cooperation OR “Community Participation”OR “Informed Consent”OR “Patient Participation”OR “Vulnerable Population” OR "Human Subject Research" OR "Human Subjects Research" OR "Community Consultation" OR recruit* OR Enrol* OR "Public Involvement" OR "public and patient involvement" OR PPI OR "patient selection" OR "Community Participation" OR "participation in biomedical research" OR "participation in Clinical Trials" OR "Health Research Participation" OR "community engagement" OR "trial participation" OR "research participation" OR "patient participation" OR "patient recruitment" OR "patient involvement" OR "patient engagement" OR "participatory research" OR "participatory action" OR Stakeholder* OR "stakeholder engagement" OR “Health Education” OR “Attitude to Health”  **1 AND 2 AND 3 AND 4**  51 results (2020) 70 (27 de agosto de 2023) |
| **GLOBAL INDEX MEDICUS**  **(comprehend AIM, LILACS, IMEMR, IMSEAR and WPRIM)**  (World Health Organization)  **Date run**:  Aug 27th, 2020 | 1. **TERMS RELATED TO TYPES OF CLINICAL RESEARCH:**   "medical research" OR "research subject" OR "participatory research" OR "biomedical research"   1. **TERMS RELATED TO COMMUNITY PARTICIPATION IN RESEARCH**   "cooperation" OR "community participation" OR "informed consent" OR "patient participation" OR "vulnerable population" OR "human subject research" OR "human subjects research" OR "community consultation" OR "recruit" OR "enrol" OR "public involvement" OR "public and patient involvement" OR "ppi" OR "patient selection" OR "community participation" OR "participation in biomedical research" OR "participation in clinical trials" OR "health research participation" OR "community engagement" OR "trial participation" OR "research participation" OR "patient participation" OR "patient recruitment" OR "patient involvement" OR "patient engagement" OR "participatory research" OR "participatory action" OR "stakeholder" OR "stakeholder engagement" OR "health education" OR "attitude to health"   1. **TERMS RELATED TO WILLINGNESS OR UNWILLINGNESS TO PARTICIPATE IN RESEARCH**   "refusal to participate" OR "motivation" OR "willingness to participate" OR "motivation to participate" OR "attitudes towards research" OR "motivate to consent" "OR motivating factor to participate" OR "willing to consent" OR "participation and retention" OR "reasons to participate" OR "facilitators to participate" OR "trial preparedness" OR "interest in participation" OR "agree to participate" OR "refusal to participate" OR "refusing participation" OR "barriers to participation" OR "barriers to participate" OR "unwilling to participate" OR "challenges to participation" OR "concerns regarding participation" OR "participants refusal" OR "refuse to join" OR "refuse to participate" OR "motivational factors" OR "knowledge and perceptions of clinical trials" OR "preparedness" OR "recruitment and retention" OR "difficult recruitment" OR "difficult to reach" OR "hard to reach"   1. **TERMS RELATED TO RESEARCH ON NEGLECTED TROPICAL DISEASES**   "buruli ulcer" OR "chagas disease" OR "dengue" OR "dracunculiasis" OR "guinea-worm disease" OR "gwd" OR "echinococcosis" OR "african trypanosomiasis" OR "trypanosomiases" OR "leishmaniasis" OR "leishmaniases" OR "leishmania infections" OR "leprosies" OR "leprosy" OR "mycetoma" OR "lymphatic filariasis" OR "elephantiasis" OR "filariasis" OR "onchocerciasis" OR "rabies" OR "lyssa" OR "hydrophobia" OR "schistosomiasis" OR "bilharziasis" OR "katayama fever" OR "schistosoma infection" OR "soil-transmitted helminthiases" OR "helminthiasis" OR "helminthiases" OR" trachoma" OR "egyptian ophthalmia" OR "teniasis" OR "taeniasis" OR "taenia infection" OR "yaws" OR "frambesia" OR "tropical medicine" OR "neglected diseases" OR "parasitic infections" OR "trematodiasis" OR "trematode infections" OR "fascioliasis" OR "clonorchiasis" OR "opisthorchiasis" OR "neurocysticercosis" OR "chikungunya" OR "snakebite envenoming" OR "snakebite" OR "snake venom" OR "scabies" OR "ectoparasitic infestations" OR "chromomycosis" OR "chromoblastomycosis" OR "cysticercosis"  **1 AND 2 AND 3 AND 4**  11 results (2020) 12 (27 de abril de 2023) |
