# Supplementary material for: Factors related to willingness to participate in biomedical research on neglected tropical diseases: A systematic review
Source: PLoS Negl Trop Dis. 2024 Mar 12;18(3):e0011996. doi: 10.1371/journal.pntd.0011996 (PMC10978008; doi:10.1371/journal.pntd.0011996)
Supplement: Attachment — Submitted filename: Response to Reviewers.docx [file pntd.0011996.s004.docx]

**Dear Nithya Venkatesan,**

I hope this letter finds you well. I would like to submit the modifications made to my manuscript titled **Factors related to willingness to participate in biomedical research on neglected tropical diseases: A Systematic Review,**which underwent peer review. I appreciate the opportunity to improve the manuscript based on the valuable suggestions and comments from the reviewers. Below are the details of the modifications made according to your observations.

With my regards,

Yours sincerely,

Mitermayer G. Reis, MD, PhD

**P1)** **Please ensure that the funders and grant numbers match between the Financial Disclosure field and the Funding Information tab in your submission form. Note that the funders must be provided in the same order in both places as well.Currently, " VRS is currently receiving a scholarship from Conselho Nacional de Desenvolvimento Científico e Tecnológico (CNPq)" is missing from the Funding Information..**

A: We have updated the information in our submission form, and we have supplemented the information in the manuscript. Now, the information is as follows: ‘This study was funded by National Institutes of Health (NIH), Grant/Award Numbers: R01 AI121330, FAPESB-PNX0001/2015 for the submitted work, and partially supported by Conselho Nacional de Desenvolvimento Científico e Tecnológico – CNPq (Proc. Proc. 306464/2016-0 and 423833/2018-9). VRS is currently receiving a scholarship from Conselho Nacional de Desenvolvimento Científico e Tecnológico (CNPq). The funders had no role in study design, data collection and analysis, decision to publish, or preparation of the manuscript.’

Top of Form

**P2) Some material included in your submission may be copyrighted. According to PLOS’s copyright policy, authors who use figures or other material (e.g., graphics, clipart, maps) from another author or copyright holder must demonstrate or obtain permission to publish this material under the Creative Commons Attribution 4.0 International (CC BY 4.0) License used by PLOS journals. Please closely review the details of PLOS’s copyright requirements here: PLOS Licenses and Copyright. If you need to request permissions from a copyright holder, you may use PLOS's Copyright Content Permission form.**

**Potential Copyright Issues:

1) [S1 Appendix] contains branding/a logo(NIHR) . We are not permitted to publish this under our CC BY 4.0 license, even with permission. We ask that you please remove or replace it.

2) [Figure2]: This Figure has been designed using images from ‘Flaticon.com’.**

A: Regarding the potential copyright issue with [S1 Appendix], we have removed the NIHR logo from the file. In addressing the potential copyright concern related to Figure 2, we replaced the 'flaticon' image with one obtained from '<https://openclipart.org/>.

We have included this attribution in the caption of Figure 2:

‘This Figure has been designed using images from ‘openclipart.org’.’

Top of Form
